# Insight Into the Effects of Nisin and Cecropin on the Oral Microbial Community of Rats by High-Throughput Sequencing

**DOI:** 10.3389/fmicb.2020.01082

**Published:** 2020-06-05

**Authors:** Lijuan Wu, Fei Li, Lisha Ran, Yanping Gao, Peijuan Xie, Jian Yang, Famin Ke, Li Liu, Qin Wang, Xiaowei Gao

**Affiliations:** ^1^Department of Endocrinology, The Affiliated Hospital of Traditional Chinese Medicine, Southwest Medical University, Luzhou, China; ^2^College of Integrated Traditional Chinese and Western Medicine, Southwest Medical University, Luzhou, China; ^3^School of Pharmacy, Southwest Medical University, Luzhou, China; ^4^Key Laboratory of Medical Electrophysiology, Ministry of Education, Institute of Cardiovascular Research, Southwest Medical University, Luzhou, China

**Keywords:** antimicrobial peptides, nisin, cecropin, oral microbiome, food additive, high-throughput sequencing

## Abstract

The oral microbiome has major impacts on oral health and disease. Antimicrobial peptides (AMPs), such as nisin and cecropin, have been widely used as food preservatives or feed additives, and are thus inevitably ingested by consumers through their oral cavity. However, as broad-spectrum antimicrobial reagents, the effect of AMPs on the oral microbiome of consumer’s remains poorly characterized. In this study, we performed 16S rDNA high-throughput sequencing to investigate the effect of nisin and cecropin on the oral microbiomes of rats. Our results suggest that although nisin and cecropin have different effects on the oral microbiome of rats, both AMPs impact the composition of oral microbial communities at the phylum and genus levels. Cecropin significantly reduced the diversity and richness of rat oral microbial communities. Notably, the relative abundance of the pathogen *Acinetobacter baumannii* increased in the oral microbial community of rats fed cecropin-containing feed. In addition, nisin significantly reduced the amount of secretory immunoglobulin A in the saliva of rats.

## Introduction

The human oral cavity contains a highly complex community of over 700 microbial species, including bacteria, viruses, fungi, and archaea (Gupta et al., 2018). Oral microbiota colonizes the oral cavity rapidly after birth to form a complex microecological system that helps maintain oral homeostasis (Zaura et al., 2014). The oral microbiome is well known to influence oral health in humans (Duran-Pinedo and Frias-Lopez, 2015). The oral microbiome plays essential roles in the development of common oral health issues, such as dental caries, periodontal diseases, and tooth loss (Gorr and Abdolhosseini, 2011; Scannapieco, 2013). Despite recent investigations, the potential association between the oral microbiome and cancer remains unclear, however, there have been reports of oral microbiome alterations in cancer patients (Bracci, 2017; Hayes et al., 2018; Healy and Moran, 2019). Thus, maintaining a normal and healthy oral microbiome is important for human health.

Antimicrobial peptides (AMPs) comprise a key component of the innate immune system of nearly all living organisms, including bacteria, plants, insects, and vertebrates (Brogden, 2005). AMPs have broad-spectrum biological activities, including anti-bacterial, anti-fungal, anti-viral, and anti-carcinogenic properties, and have been considered as excellent alternatives to antibiotics (Pfalzgraff et al., 2018). To date, over 2,600 AMPs have been identified, several of which have been successfully applied in the medical and food industries (da Silva Malheiros et al., 2010; Yi et al., 2014). In humans, over 45 AMPs from different functional classes are secreted into the oral cavity by oral epithelial cells, neutrophils, and salivary glands (Gorr and Abdolhosseini, 2011). Theses AMPs are thought to protect the oral epithelia from various pathogenic microbes and maintain oral homeostasis of commensal bacteria. Thus, AMPs have a remarkable influence on the composition of oral microbial communities and play an important role in oral health.

Nisin and cecropin are two agriculturally important AMPs. Nisin is produced by the probiotic bacterium *Lactococcus lactis* and has antimicrobial activities against gram-positive bacteria, including foodborne pathogens such as *Listeria monocytogenes* (Tong et al., 2010). Mature nisin is generated by post-translationally modifying an immature precursor to produce a polypeptide with 34 amino acid residues containing one lanthionine, four methyl-lanthionine rings, and unusual residues such as dehydroalanine and dehydrobutyrine (Tong et al., 2010). Nisin has been granted a “generally regarded as safe” (GRAS) status and is approved for use as food preservative by the World Health Organization (WHO), the European Food Safety Authority (EFSA), and the United States Food and Drug Administration (FDA) (Szendy et al., 2019). Currently, it is widely used in dairy products and canned foods (Ahmad et al., 2017). Cecropin was the first AMP isolated from insects and is derived from the hemolymph of the moth *Hyalophora cecropia* (Hancock and Sahl, 2006). Members of the cecropin family typically have peptide chains consisting of 31–39 amino acids and possess broad-spectrum antimicrobial activities (Brady et al., 2019). Several studies have shown that cecropin, when used as a feed additive, can increase the performance of livestock and poultry while also protecting them from lethal pathogen infections (Wen and He, 2012; Wu et al., 2012; Xiao et al., 2015; Shrestha et al., 2019).

The use of exogenous AMPs as food preservatives or additives may have little impact on the gut microbiome of consumers because they are readily digested by proteolytic enzymes in the digestive system (Gough et al., 2018). However, during dietary consumption by humans or animals, these AMPs would first pass through the oral cavity, and there is little known about their potential effects on the oral microbiome. In this study, we investigated the effects of nisin and cecropin on the oral microbial communities of rats using 16S rDNA high-throughput sequencing. Rats fed cecropin-containing feed showed significant differences in their oral microbiome compositions compared with the control ones. Specifically, cecropin significantly decreased both the diversity and richness of the oral microbial community. Additionally, the amount of oral secretory immunoglobulin A (sIgA) was significantly lower in the group fed nisin-containing feed than in the control group. Our results suggest that nisin and cecropin have different effects on the composition of the oral microbiome and oral immune system of rats. Our results provide insight into the influence of exogenous AMPs in food on the oral microbiome, which represents one of the most complex microecological systems in humans and animals.

## Materials and Methods

### Reagents and Experimental Diets

Commercially available nisin (amino acid sequence: I T S I S L C T P G C K T G A L M G C N M K T A T C H C S I H V S K) and cecropin (amino acid sequence: K W K L F K K I E K V G Q N I R D G I I K A GP A V A V V G Q A T Q I A K) were purchased from Hengkang Food Additive Co., Ltd. (Tianjin, China) and Shandong Ruitai Biotechnology Co., Ltd. (Shandong, China), respectively. A commercially formulated diet for rats was selected as the basal control diet that consisted of 46.74% cornmeal, 12% fish meal, 26% soybean meal, 10% flour, 3% soybean oil, 0.2% methylcellulose, 1% salt, 0.26% methionine, and 0.8% vitamin and mineral mixture. Experimental diets were prepared by adding 0.05% nisin or 0.01% cecropin powder directly to the basal control diet during feed preparation. The concentrations of nisin and cecropin used in this study are the working concentrations recommended by manufacturers during common use for food preservation and as feed additives.

### Feeding Experiment

This study was approved by the Southwest Medical University Ethics Committee (approval number: 201903-165). Thirty 4-week-old male rats were purchased from the experimental animal base of the Southwest Medical University (Luzhou, China). Rats were weighed and randomly divided into three groups (10 rats per group). All rats were fed the control diet for 1 week to acclimatize them to the environment before beginning experiments. During the experiments, the two test groups were fed experimental diets containing nisin or cecropin, while the control group was fed the control diet. Rats were maintained in a 12:12 h light–dark cycle and weighed weekly after beginning the experiment.

### ELISA Analysis

At 4 weeks, saliva samples were collected from the hypoglottis of rats using a microsampler and used for ELISA analysis. The amount of sIgA in the samples was measured using a Rat sIgA ELISA kit (Lilai Biotechnology Co., Ltd., Chengdu, China) according to the manufacturer’s instructions. Briefly, the saliva samples and standards were incubated with an anti-rat sIgA antibody immobilized in 96-well plates at 37°C for 1 h. After washing three times with wash buffer, a specific biotinylated antibody was added to the wells to bind the immobilized proteins. Then, streptavidin-conjugated horseradish peroxidase was added to bind the biotinylated antibody. Colorimetric substrate was added to proportionally convert the amount of horseradish peroxidase–streptavidin–biotin conjugate in the well into visible color. Colorimetric signals (OD450 nm) were measured with a plate reader. The final concentrations of sIgA per well were calculated based on the standard curve.

### DNA Extraction and PCR Amplification

Saliva and supragingival biofilm samples were collected from the oral cavity of experimental and control rats using sterile oral swabs and then used for oral microbiota community analysis. Prior to sample collection, the area surrounding the oral cavity of each rat was sterilized with 70% ethanol. Microbial DNA was extracted from oral swabs using the E.Z.N.A. Stool DNA kit (OMEGA, Bio-tek, United States) according to the manufacturer’s instructions. Briefly, oral swab samples were transferred to 2 ml sterile tubes containing 200 mg of glass beads. After adding 300 μl of SP1 buffer and 10 μl of Proteinase K solution, the tubes were vortexed for 10 min then incubated at 70°C for 15 min (samples were additionally incubated at 90°C for 5 min as needed). After cooling on ice, 100 μl of SP2 buffer and 200 μl of HTR were added to the samples. After centrifugation, DNA was extracted from the resulting supernatants. DNA quality was analyzed by 1% agarose gel electrophoresis. The concentrations of microbial DNA were determined using a NanoDrop 2000 UV-vis spectrophotometer (Thermo Scientific, Wilmington, CA, United States).

Using the extracted genomic DNA as a template, the V3/V4 region of the 16S rRNA gene was amplified with the following primers: 338F (5′-ACTCCTACGGGAGGCAGCAG-3′) and 806R (5′-GGACTACHVGGGTWTCTAAT-3′). Amplification of the 16S rRNA sequence was performed in a 25-μl PCR mixture containing 9 μl of ddH_2_O, 2.5 μl of KOD-Plus-Neo PCR buffer (10×), 1 μl of 25 mM MgSO_4_, 1 μl each of 10 μM 338F and 806R primers, 2.5 μl of 2 mM dNTPs, 1 μl of KOD-Plus-Neo DNA polymerase (1 U/μl), and 2 μl of genomic DNA template (∼10 ng). PCR conditions were as follows: initial denaturation at 94°C for 5 min, 30 cycles of 94°C for 30 s (denaturing), 55°C for 30 s (annealing), 68°C for 30 s (extension), and a final extension at 68°C for 5 min. After amplification, the PCR products were visualized by 1% agarose gel electrophoresis and purified using the DNA Gel Extraction Kit (OMEGA, Bio-tek, United States).

### Illumina MiSeq Sequencing

Prior to sequencing, DNA quality was analyzed on an Agilent 2100 Bioanalyzer (Agilent, United States), and concentrations were determined using a QuantiFluor^TM^-ST Fluorescence Quantitative System (Promega Company, United States). After quantification, DNA was mixed in equimolar ratios according to the sequencing requirements. Paired end (PE) libraries were constructed by PCR using the TruSeq^TM^ DNA Sample Prep Kit (Illumina). Illumina MiSeq sequencing was performed at Majorbio Bio-Pharm Technology Co., Ltd. (Shanghai, China).

### Sequence Processing and Bioinformatics Analysis

The resulting raw sequences obtained by MiSeq sequencing were processed and quality-filtered using the FLASH and Trimmomatic software with the following criteria: (a) reads with a quality score < 20 were removed; (b) reads containing ambiguous bases were removed; (c) PE reads containing overlapping sequences longer than 10 bp were merged into one sequence; (d) the maximum mismatch ratio allowed in the overlap area of spliced sequences was 0.2; and (e) samples were distinguished by barcode and primers. The maximum allowed number of mismatches in barcodes and primers is 0 and 2, respectively.

To estimate the bacterial community diversity in different samples, we performed operation classification unit (OTU) analysis (Rosselló-Mora and Amann, 2001). Valid sequences with more than 97% similarity were defined as one OTU, and chimeric sequences were identified and removed using USEARCH version 7.0^1^. Data were analyzed on the Majorbio i-Sanger Cloud Platform^2^. The number of OTUs and sequences in each OTU were calculated using the i-Sanger Cloud Platform. Rarefaction curves generated by plotting the number of OTUs against the number of identified sequences were used to estimate if the sequences obtained from each sample were sufficient for species identification. α-Diversity analysis was performed using the Mothur software to calculate the Sobs, Chao, Ace, Shannon, and Simpson indices of the three experimental groups (Schloss et al., 2011). β-Diversity analysis was performed to examine the community diversity and species differences among the three groups. Principal coordinate analysis (PCoA) and hierarchical clustering trees were completed using an R software package. To determine if there were statistically significant differences in microbial composition across the three groups, non-parametric one-way analysis of variance (ANOVA; Kruskal–Wallis) was performed, and *p* < 0.05 was considered statistically significant. A hierarchical clustering heatmap analysis was also carried out to identify the main differences in species among the three experimental groups.

### Statistical Analysis

The data in this study were analyzed by one-way ANOVA using SPSS version 20.0. A value of *p* < 0.05 was considered statistically significant.

## Results

### Effects of Nisin and Cecropin on Growth

**FIGURE 1:**
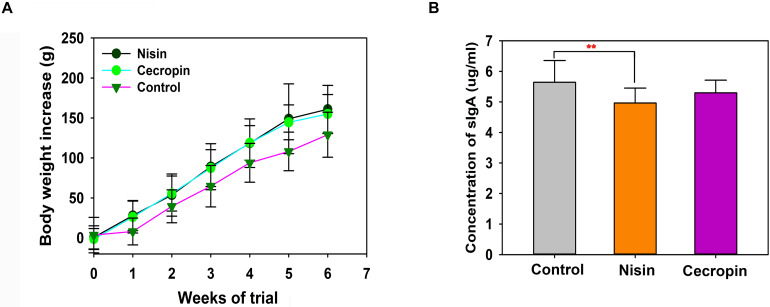
Effects of nisin and cecropin on rat body weight and oral immune system. **(A)** Cumulative weight gain of rats in each group. Rats were weighed weekly after beginning the experiment. Weight gain was calculated for each rat using the following equation: Weight gain = final weight – initial weight. **(B)** Concentrations of secretory immunoglobulin A (sIgA) in the saliva of rats in the three groups. *p* < 0.05 was considered statistically significant (**p < 0.01).

Figure 1A shows the effects of nisin and cecropin on the growth of rats. Rats fed nisin or cecropin did not show any differences in body weight gain compared with each other, however, both groups exhibited slightly higher body weight gain than the control group. At the fourth week, the nisin and cecropin groups had approximately 26.8% more body weight gain than the control group. This suggests that nisin and cecropin in feed may have positive effects on rat growth.

### Effects of Nisin and Cecropin on the Oral Immune System

To determine the effects of nisin and cecropin on the oral immune system of rats, the concentration of sIgA in oral samples was determined by ELISA. Rats in the nisin group had significantly lower sIgA concentrations than the control group (Figure 1B), while the cecropin group was not significantly different from the control group. These results suggest that adding nisin to feed reduces the amount of sIgA in the oral saliva of rats.

### Effects of Nisin and Cecropin on the Oral Microbiome

**FIGURE 2:**
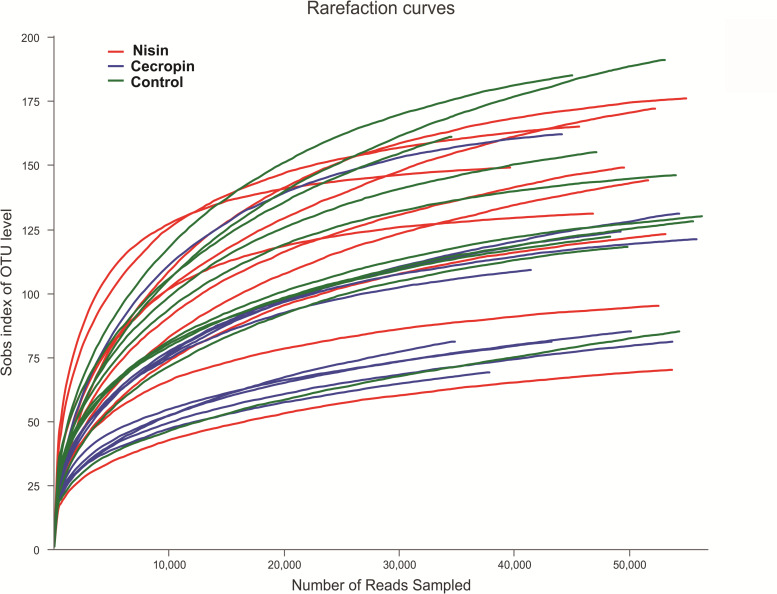
Rarefaction analysis of operation classification units (OTUs) in different samples. Total sample richness is presented as the richness estimator Sobs index. The curve was generated by plotting the number of valid sequences for each rat against the Sobs index of OTUs.

The sequences obtained in this study were deposited into the NCBI short read archive database (accession number: PRJNA601865). After removing low-quality reads, 1,527,561 valid sequences were obtained from the 30 samples. These sequences were delineated into 762 OTUs with a 97% sequence similarity cut-off threshold (Supplementary Table S1). Sequence Good’s coverage was ≥99% in all 30 samples (Supplementary Table S2). The rarefaction curve of each sample tended to reach the saturation plateau (Figure 2). Together, these results suggest that sufficient sampling depth was obtained, and the majority of bacteria phylotypes present in the samples could be identified.

#### Microbial Community Diversity Analysis

**FIGURE 3:**
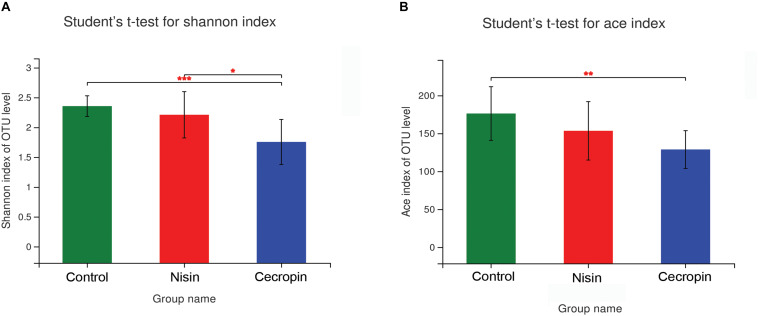
The α-diversity of oral microbial communities is significantly different between the three experimental groups. **(A)** Bacterial community diversity presented as the Shannon index. **(B)** Bacterial community richness presented as the ACE index. *p* < 0.05 was considered statistically significant (*p < 0.05, **p < 0.01, ***p < 0.005).

To determine the effects of nisin and cecropin on oral microbial community diversity, the α-diversity and β-diversity of the microbial communities were analyzed separately. For α-diversity analysis, the richness index (ACE) and community diversity index (Shannon) of the three experimental groups were calculated and compared. The Shannon index was significantly lower in the cecropin group than in the control and nisin groups, suggesting that the oral microbial community diversity of rats decreased after consuming cecropin-containing feed, but not after consuming nisin-containing feed (Figure 3A). The ACE index was lower in both the cecropin and nisin groups than in the control group. Additionally, the ACE index of the cecropin group was significantly lower than that of the nisin or control groups (Figure 3B). This suggests that nisin and cecropin can reduce the species richness of the oral microbiota in rats.

**FIGURE 4:**
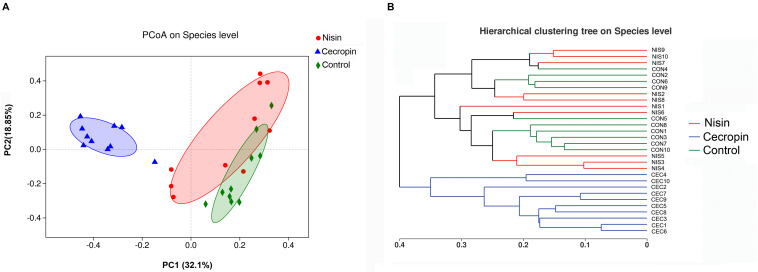
Relationship of bacterial community compositions in different rat samples. **(A)** Principal coordinate analysis (PCoA) of different bacterial communities. Principal component 1 (PC1) and 2 (PC2) explained 32.1 and 18.85% of the variance, respectively. Distances between each symbol in the plot reflect relative dissimilarities of relative bacterial communities. **(B)** Hierarchical cluster tree of Bray–Curtis distances. Each branch represents one rate oral bacterial community.

Principal coordinate analysis and Bray–Curtis distance analyses were performed to assess the β-diversity of the microbial communities. The PCoA score plot was determined using the weighted UniFrac distance, and each symbol on the plot represents one sample (Figure 4A). According to the PCoA score plot, the cecropin group showed structural differences compared with the control group, whereas the nisin group showed only slight dissimilarity with the control group. The two principal coordinate axes accounted for 50.59% of the total variation among the three groups. A hierarchical clustering tree is shown in Figure 4B where each branch on the tree represents the oral microbiota community of one sample. The nisin group clustered together with the control group, and the cecropin group is located on a different branch of the tree. Taken together, these results suggest that consuming cecropin-containing feed restructures the oral microbial communities of rats.

#### Microbial Community Composition Analysis

**FIGURE 5:**
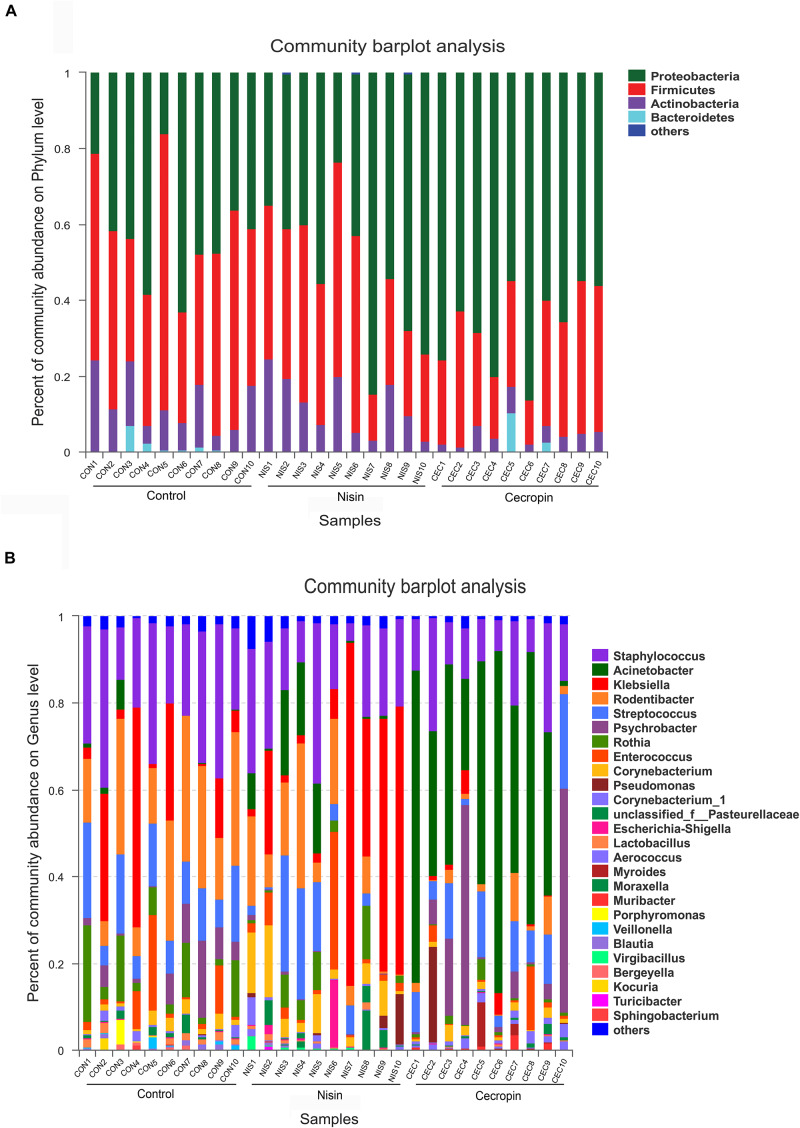
Bacterial community compositions of different rat samples. Relative abundance of bacteria at the phylum **(A)** and genus **(B)** levels. “Others” refers to bacteria with less than 1% abundance at the genus and phylum levels.

Based on the phylogenetics information, all sequences obtained from the 30 samples were assigned to 23 bacterial phyla using the program Mothur with default settings. The number of phyla in the nisin, cecropin, and control groups were 23, 16, and 16, respectively. At the phylum level, the three groups showed similar 16S rRNA profiles of abundant sequences, and Proteobacteria, Firmicutes, and Actinobacteria represent the three major phyla observed (Figure 5A). The relative abundance of Bacteroidetes was also greater than 1% in some samples from the control and cecropin groups.

**FIGURE 6:**
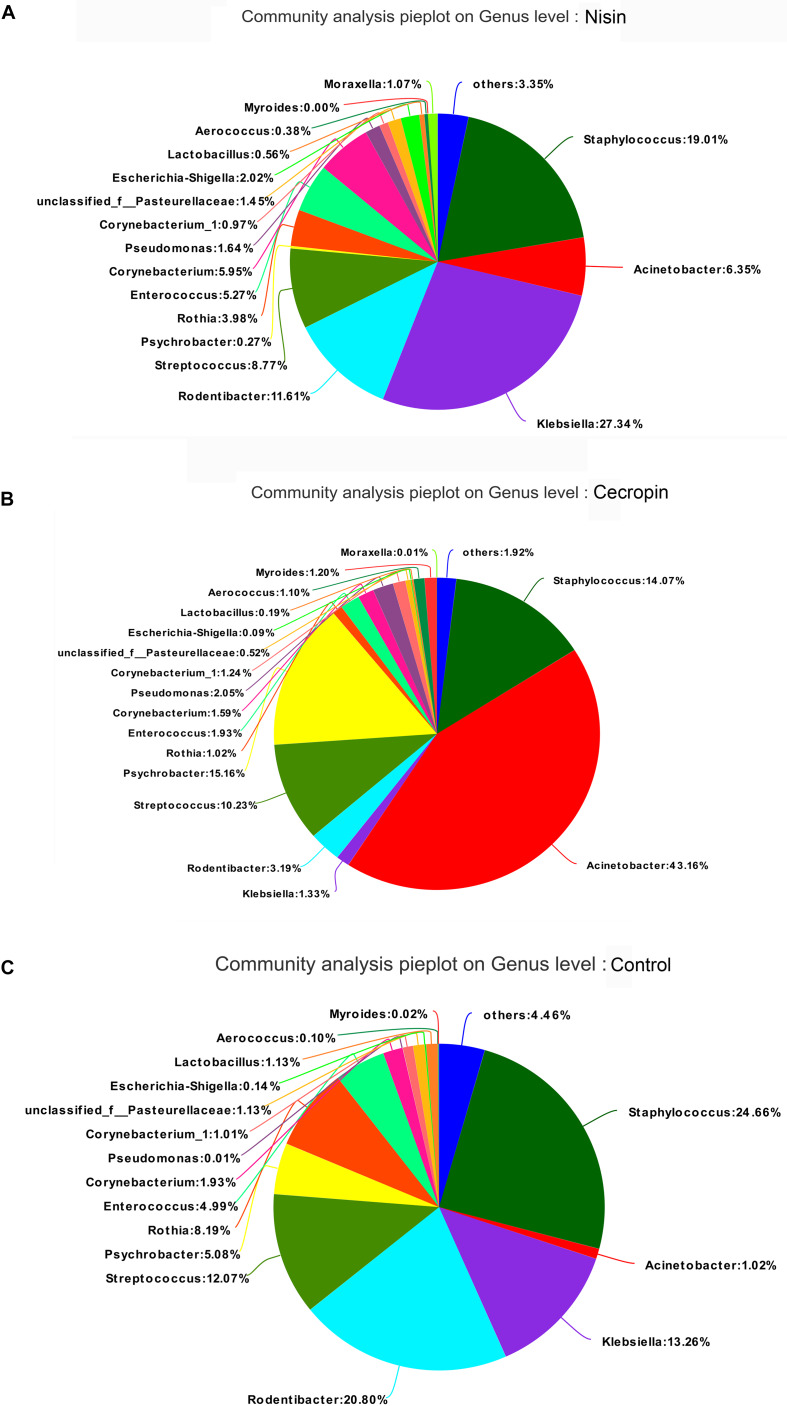
Distribution of bacterial genera among the three groups. Pie diagrams show the bacterial composition of the nisin group **(A)**, cecropin group **(B)**, and control group **(C)**. The relative abundance of bacterial genera in each group are shown. “Others” refers to bacteria with less than 1% abundance at the genus level.

The relative abundance of each bacterial genus is shown in Figure 5B. A total of 450 different genera were identified from the 30 samples, and only 26 genera had a relative abundance greater than 1%. The five most abundant genera in the control group were *Staphylococcus* (24.66%), *Rodentibacter* (20.80%), *Klebsiella* (13.26%), *Streptococcus* (12.07%), and *Rothia* (8.19%), whereas the top five genera in the cecropin group were *Acinetobacter* (43.16%), *Psychrobacter* (15.16%), *Staphylococcus* (14.07%), *Streptococcus* (10.23%), and *Rodentibacter* (3.19%) (Figure 6). The five most abundant genera in the nisin group were *Klebsiella* (27.34%), *Staphylococcus* (19.01%), *Rodentibacter* (11.61%), *Acinetobacter* (6.35%), and *Corynebacterium* (5.95%). These results suggest that the three groups show differences in the abundance of genera present in the oral bacterial community.

#### Overall Changes of Oral Microbiome Caused by Nisin and Cecropin

**FIGURE 7:**
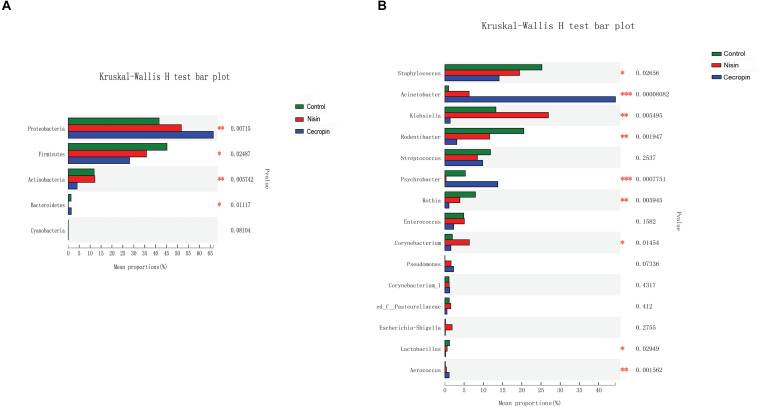
Kruskal–Wallis analysis of the differences in specific microbial taxa among the three groups. **(A)** Differences at the phylum level. **(B)** Differences at the genus level (*p < 0.05, **p < 0.01, ***p < 0.005).

Using the Kruskal–Wallis analysis, significant variations in oral microbiome composition were observed in the nisin and cecropin groups at the phylum and genus levels when compared with the control group (Figure 7). At the phylum level, significant differences were detected in the relative abundance of Proteobacteria, Firmicutes, Actinobacteria, and Bacteroidetes among the three groups. The relative abundance of Proteobacteria increased, whereas that of Firmicutes decreased in the nisin and cecropin groups compared with that in the control group. Further, the cecropin group showed greater changes in the relative abundance of these two phyla than the nisin group. The relative abundance of Actinobacteria in the cecropin group and Bacteroidetes in the nisin group was significantly lower than in the control group.

**FIGURE 8:**
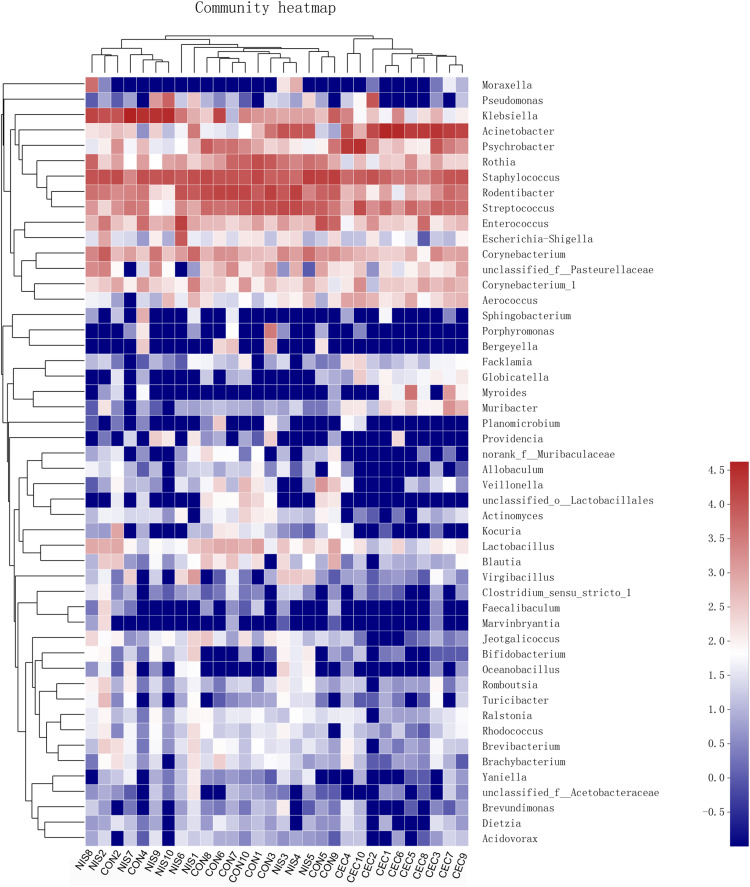
Heatmap of oral bacteria genera from the three groups. The relationship among different rats (top) was calculated by Bray distance and the complete clustering method. The bacterial phylogenetic tree (left) was determined using the neighbor-joining method. The relative abundance values of bacteria genera in different samples are depicted by color intensity; right: the color legend corresponding to different values.

As shown in Figure 7B, statistically significant differences in the relative abundance of nine genera were detected among the three groups. The abundance of *Staphylococcus*, *Rodentibacter*, *Rothia*, and *Lactobacillus* was significantly lower, whereas that of *Acinetobacter*, *Aerococcus*, and *Pseudomonas* was increased in the nisin and cecropin groups compared with that in the control. In addition, when compared with the control group, the relative abundance of *Klebsiella* was increased in the nisin group but decreased in the cecropin group, whereas the proportion of *Psychrobacter* was lower in the nisin group but higher in the cecropin group. At the species level, the relative abundance of the opportunistic pathogen *Acinetobacter baumannii* was strikingly increased and became the dominant species in the cecropin group (Supplementary Figure S1). The proportion of *Pseudomonas aeruginosa* was also increased in the cecropin group. The abundance of *Klebsiella variicola* was increased in the nisin group but decreased in the cecropin group. These results suggest that some pathogens exhibit increased abundance in the oral microbiome of rats fed nisin- or cecropin-containing feeds. Hierarchical clustering heatmap analysis was performed at the genus level to reveal changes in the entire microbial community structure caused by nisin and cecropin. As shown in Figure 8, the composition of the oral microbiota in the nisin group was more similar to that of the control group when compared with the cecropin group. Taken together, these results demonstrate that both nisin and cecropin change the composition of oral microbial communities of rats. In addition, more striking changes are observed with cecropin than with nisin.

## Discussion

Oral health depends on a normal and healthy oral microbiome (Scannapieco, 2013). To build a stable oral microbiome, the oral microbiota must combat the daily physical and chemical perturbations associated with food consumption. Many endogenous and exogenous factors, including food habits, hormonal imbalance, stress, puberty, poor oral hygiene, tobacco and alcohol consumption, diabetes, and fluctuations in temperature, pH, antimicrobial, and dietary components, can affect the composition of oral microbial communities. The loss of homeostasis between commensal and pathogenic bacteria may result in oral diseases, such as dental caries and periodontal disease. Dental caries is a polymicrobial disease caused by a dental plaque biofilm composed of *Lactobacillus* spp., *Streptococcus* spp., *Actinomyces* spp., and many other microbes (Scannapieco, 2013). *Streptococcus mutans* and related species (*S. sobrinus*, *S. pyogenes*, *S. cricetus*, *S. rattus*, *S. downei*, and *S. macacae*) have long been considered to be the main etiological agents of dental caries. However, more recent studies using molecular approaches have demonstrated that additional species belonging to the genera *Lactobacillus*, *Atopobium*, and *Propionibacterium* are also present in carious lesions at significantly higher levels than previously thought (Krishnan et al., 2017). Periodontal diseases result from the host response to biologically active products (e.g., chemotactic peptides, organic acids, lipopolysaccharide, and protein toxins) produced by the tooth biofilm. A small number of anaerobic species, including *Porphyromonas gingivalis*, *Tannerella forsythia*, *Treponema denticola*, contribute to the etiology of this disease (Scannapieco, 2013).

Nisin and cecropin are two widely known AMPs with extensively explored antimicrobial activities. Nisin has been shown to inhibit many oral microbiota species *in vitro*, including *Streptococcus sanguinis*, *S. gordonii*, *S. mutans*, *S. sobrinus*, *Lactobacillus acidophilus*, *L. casei*, *L. fermenti*, *Actinomyces viscosus*, and *A. naeslundii* (Tong et al., 2010; da Silva et al., 2012). The antimicrobial activities of nisin are not affected by the chemical environment of the oral cavity (including enzymes, proteins, and other inorganic components in the saliva). Some oral bacterial species, such as *S. mutans*, *S. sobrinus*, and *L. acidophilus*, can ferment carbohydrates into acid products to maintain a low pH in dental plaque that is conducive to the antibacterial activity and stability of nisin (Tong et al., 2014). The *in vivo* effects of nisin on oral cavity microflora have been investigated in a culture-dependent manner, but no significant differences were observed (Cowell et al., 1971). It should be noted that traditional culture-based methods may not reveal all structural changes in the oral microbiota because some oral microbes affected by nisin may not be culturable. Non-traditional molecular-based approaches, such as metagenomics and next-generation sequencing techniques, should be used to comprehensively reveal all changes in the oral microbiota caused by nisin and cecropin. In the present study, we used 16S rDNA high-throughput sequencing to reveal the effects of nisin and cecropin on the oral microbiome of rats. Our results clearly show that nisin and cecropin affect the diversity and composition of the rat oral microbiota.

Both the community diversity index (Shannon) and the richness index (ACE) significantly decreased in the cecropin group, suggesting that the diversity and richness of the oral microbial community of rats decreased after consuming cecropin-containing feed (Figure 3). Thus, cecropin inhibits the oral microbiota of rats when used as a feed additive. The PCoA plot and hierarchical clustering tree revealed that the microbial communities of the three groups were divided into two distinct clusters. The cecropin group clustered away from the nisin and control groups, while the nisin group partially overlapped with the control group in the PCoA plot and was located in the same branch of the tree (Figure 4). Taken together, we conclude that nisin and cecropin have different effects on the oral microbial community diversity of rats. We speculate that these differences may be attributed to different antimicrobial spectrums and mechanisms of action between the two AMPs (Ahmed and Hammami, 2019).

In the present study, the dominant phyla of the oral microbiome in rats were Proteobacteria, Firmicutes, Actinobacteria, and Bacteroidetes. This result is consistent with a previous study that showed the same major phyla in rat oral samples (Hyde et al., 2014). Nisin and cecropin changed the composition of the oral microbiome at both phylum and genus levels (Figure 7). Nisin and cecropin reduced the relative abundance of *Staphylococcus* in the oral microbial community. Some *Staphylococcus* spp. are potential pathogens and are closely associated with the occurrence of pyogenic diseases. Compared with the control group, the genus *Klebsiella* was reduced in the cecropin group and significantly increased in the nisin group. *Klebsiella* spp. is a causal pathogen of many diseases, including peritonitis, diarrhea, sepsis, pneumonia, meningitis, respiratory tract infections, and urinary tract infections. Both nisin and cecropin significantly reduced the relative abundance of *Rodentibacter* in the oral community of rats. Similarly, nisin and cecropin reduced the richness of the genus *Rothia*. *Rothia* spp. can induce infectious endocarditis in humans (Schafer et al., 1979). The abundance of *Psychrobacter* was significantly decreased in the nisin group. This result is unexpected given that members of this genus are gram negative and should be more tolerant to nisin, which is typically active against gram-positive bacteria (Batpho et al., 2017). Further studies are needed to clarify this discrepancy. Based on these results, nisin and cecropin alter the abundance of some pathogens and opportunistic pathogens in the oral microbial community of rats. However, it should be noted that these pathogens or opportunistic pathogens are not present in the normal oral flora of humans, and *in vivo* investigations are needed to clarify whether nisin and cecropin can also affect the abundance of oral pathogens of humans.

The proportion of the genus *Acinetobacter* was roughly 43 times higher in the cecropin group than in the control group. At the species level, this extraordinary increase observed in the cecropin group was mainly caused by the increase in *A. baumannii*, one of the most common bacterial species involved in hospital infections. *A. baumannii* causes a wide range of infections, including pneumonia and bloodstream infections (Dijkshoorn et al., 2007). This result may indicate that cecropin is not active against *A. baumannii*, and *A. baumannii* may then occupy the niche of other oral microbiota species that were inhibited by cecropin to become the dominant species in the oral microbiome of cecropin-exposed rats. However, growth of *A. baumannii* may be inhibited in the control group by commensal bacteria present in the oral cavity.

Although nisin displayed weaker effects on the oral microbiota community than cecropin, it significantly reduced sIgA content in the saliva of rats. Further studies should be performed to reveal the potential mechanisms underlying this phenomenon. Given that AMPs are typically the effector molecules of innate and adaptive immunity capable of modulating pro- and anti-inflammatory responses, and have chemotactic activity, nisin may directly regulate the oral mucosal immune responses of rats (Lai and Gallo, 2009; Mansour et al., 2014). Nisin may also reduce sIgA expression indirectly by altering the oral microbiota composition, which is known to affect oral mucosal immunity (Zaura et al., 2014).

It is widely accepted that oral diseases in humans are primarily caused by oral microorganisms in a synergistic or cooperative manner (Zaura et al., 2014). In addition to the host immune response, the dynamic balance of synergistic and antagonistic interspecies interactions in the microbial community determines whether or not disease occurs (He et al., 2015). In the present study, nisin and cecropin changed the diversity and composition of the oral microbiome of rats, reduced the relative abundance of some specific pathogens, and increased the abundance of other pathogens. The oral microbiota composition of rats differs from that of humans, and the alterations in the oral microbiome of rats observed in this study cannot be directly applied to human oral microbiota. However, by performing *in vitro* or *in vivo* experiments, several studies have demonstrated that nisin and cecropin can inhibit the growth and proliferation of human oral pathogens (Moore et al., 1996; Tong et al., 2014). Based on a clinical study, (Noordin and Kamin, 2007) demonstrated that nisin can reduce plaque accumulation and gingivitis by inhibiting the growth and proliferation of oral microorganisms in the human oral cavity. Turner et al. (2004) investigated the effects of nisin on *Enterococcus faecalis* and *S. gordonii*, and found that nisin may effectively eliminate these species from the root canal system. Thus, nisin and cecropin have shown great potential to affect the composition of human oral microbiota. Considering that nisin and cecropin have been widely used as food preservatives or feed additives, their long-term effects on the oral microbiome and oral health of humans and other consumers (poultry and livestock) should be more carefully studied.

## Data Availability Statement

The datasets generated for this study can be found in the NCBI BioProject under accession number PRJNA601865 (https://www.ncbi.nlm.nih.gov/bioproject/PRJNA601865/) and also available on request to the corresponding author.

## Ethics Statement

The animal study was reviewed and approved by the Southwest Medical University Ethics Committee.

## Author Contributions

XG and QW conceived and designed the experiments. LW, FL, LR, YG, PX, and JY performed the experiments. XG, LW, LL, and FK analyzed the data. XG wrote the initial draft of the manuscript. LL and QW critically revised the manuscript. All authors have read and approved the final manuscript.

## Conflict of Interest

The authors declare that the research was conducted in the absence of any commercial or financial relationships that could be construed as a potential conflict of interest.

## Footnotes

1 http://drive5.com/uparse/

2 www.i-sanger.com
