# Supplementary material for: Insight Into the Effects of Nisin and Cecropin on the Oral Microbial Community of Rats by High-Throughput Sequencing
Source: Front Microbiol. 2020 Jun 5;11:1082. doi: 10.3389/fmicb.2020.01082 (PMC7292207; doi:10.3389/fmicb.2020.01082)
Supplement: Supplementary file 1 [file Data_Sheet_1.docx]

**Table S1. Information of valid reads of 30 samples in this study.**

| Sample | Sequence number | Base number | Mean length | Minimal length | Maximal length |
| --- | --- | --- | --- | --- | --- |
| NIS1 | 51565 | 21974540 | 426 | 402 | 432 |
| NIS2 | 46542 | 19800149 | 425 | 388 | 431 |
| NIS3 | 58070 | 24765426 | 426 | 214 | 441 |
| NIS4 | 53918 | 23068659 | 427 | 393 | 432 |
| NIS5 | 52492 | 22345159 | 425 | 372 | 526 |
| NIS6 | 56084 | 24007569 | 428 | 253 | 432 |
| NIS7 | 54145 | 23198279 | 428 | 262 | 431 |
| NIS8 | 50903 | 21664798 | 425 | 254 | 431 |
| NIS9 | 47764 | 20418656 | 427 | 402 | 431 |
| NIS10 | 57225 | 24519030 | 428 | 369 | 456 |
| CEC1 | 52119 | 22375421 | 429 | 222 | 431 |
| CEC2 | 49138 | 21083549 | 429 | 222 | 431 |
| CEC3 | 55810 | 23898927 | 428 | 262 | 432 |
| CEC4 | 44411 | 19030143 | 428 | 385 | 431 |
| CEC5 | 40343 | 17250785 | 427 | 369 | 482 |
| CEC6 | 44003 | 18894431 | 429 | 392 | 431 |
| CEC7 | 49656 | 21277233 | 428 | 403 | 438 |
| CEC8 | 36244 | 15547242 | 428 | 255 | 432 |
| CEC9 | 42116 | 18048666 | 428 | 403 | 432 |
| CEC10 | 56288 | 24097227 | 428 | 403 | 431 |
| CON1 | 54984 | 23322975 | 424 | 262 | 431 |
| CON2 | 38087 | 16249995 | 426 | 369 | 431 |
| CON3 | 60551 | 25752658 | 425 | 242 | 476 |
| CON4 | 62086 | 26569782 | 427 | 204 | 455 |
| CON5 | 50843 | 21713112 | 427 | 403 | 431 |
| CON6 | 57485 | 24586285 | 427 | 345 | 456 |
| CON7 | 47855 | 20360625 | 425 | 389 | 431 |
| CON8 | 46353 | 19838770 | 427 | 392 | 435 |
| CON9 | 60476 | 25858589 | 427 | 270 | 431 |
| CON10 | 50005 | 21285809 | 425 | 392 | 431 |

**Table S2. Table 1. Richness and diversity indexes as calculated by MOTHUR software and estimated sample coverage for 16S rRNA libraries of different samples**

| Sample | sobs | shannon | simpson | ace | chao | Coverage (%) |
| --- | --- | --- | --- | --- | --- | --- |
| NIS1 | 149 | 2.77 | 0.11 | 152 | 152 | 99.97 |
| NIS2 | 165 | 2.70 | 0.11 | 172 | 171 | 99.96 |
| NIS3 | 95 | 1.99 | 0.21 | 102 | 99 | 99.97 |
| NIS4 | 172 | 2.38 | 0.15 | 209 | 194 | 99.92 |
| NIS5 | 144 | 2.08 | 0.19 | 179 | 173 | 99.92 |
| NIS6 | 176 | 2.31 | 0.14 | 184 | 183 | 99.96 |
| NIS7 | 123 | 1.52 | 0.34 | 135 | 137 | 99.95 |
| NIS8 | 149 | 2.40 | 0.13 | 175 | 174 | 99.93 |
| NIS9 | 131 | 2.11 | 0.19 | 134 | 136 | 99.97 |
| NIS10 | 70 | 1.80 | 0.24 | 86 | 85 | 99.96 |
| CEC1 | 85 | 1.22 | 0.53 | 129 | 108 | 99.95 |
| CEC2 | 62 | 2.01 | 0.19 | 110 | 81 | 99.95 |
| CEC3 | 131 | 1.93 | 0.26 | 163 | 157 | 99.93 |
| CEC4 | 162 | 1.79 | 0.31 | 170 | 168 | 99.95 |
| CEC5 | 69 | 1.83 | 0.29 | 91 | 90 | 99.94 |
| CEC6 | 81 | 1.08 | 0.62 | 108 | 102 | 99.94 |
| CEC7 | 124 | 2.21 | 0.19 | 144 | 143 | 99.94 |
| CEC8 | 81 | 1.53 | 0.41 | 115 | 110 | 99.92 |
| CEC9 | 109 | 2.18 | 0.20 | 121 | 118 | 99.95 |
| CEC10 | 121 | 1.73 | 0.32 | 131 | 129 | 99.96 |
| CON1 | 146 | 2.38 | 0.13 | 153 | 151 | 99.97 |
| CON2 | 161 | 2.63 | 0.12 | 201 | 195 | 99.86 |
| CON3 | 130 | 2.31 | 0.16 | 144 | 144 | 99.95 |
| CON4 | 85 | 2.06 | 0.18 | 215 | 133 | 99.93 |
| CON5 | 118 | 2.13 | 0.16 | 133 | 126 | 99.95 |
| CON6 | 128 | 2.39 | 0.14 | 152 | 147 | 99.94 |
| CON7 | 155 | 2.25 | 0.16 | 174 | 173 | 99.93 |
| CON8 | 185 | 2.39 | 0.15 | 205 | 200 | 99.92 |
| CON9 | 191 | 2.55 | 0.11 | 234 | 236 | 99.90 |
| CON10 | 122 | 2.41 | 0.15 | 146 | 147 | 99.94 |


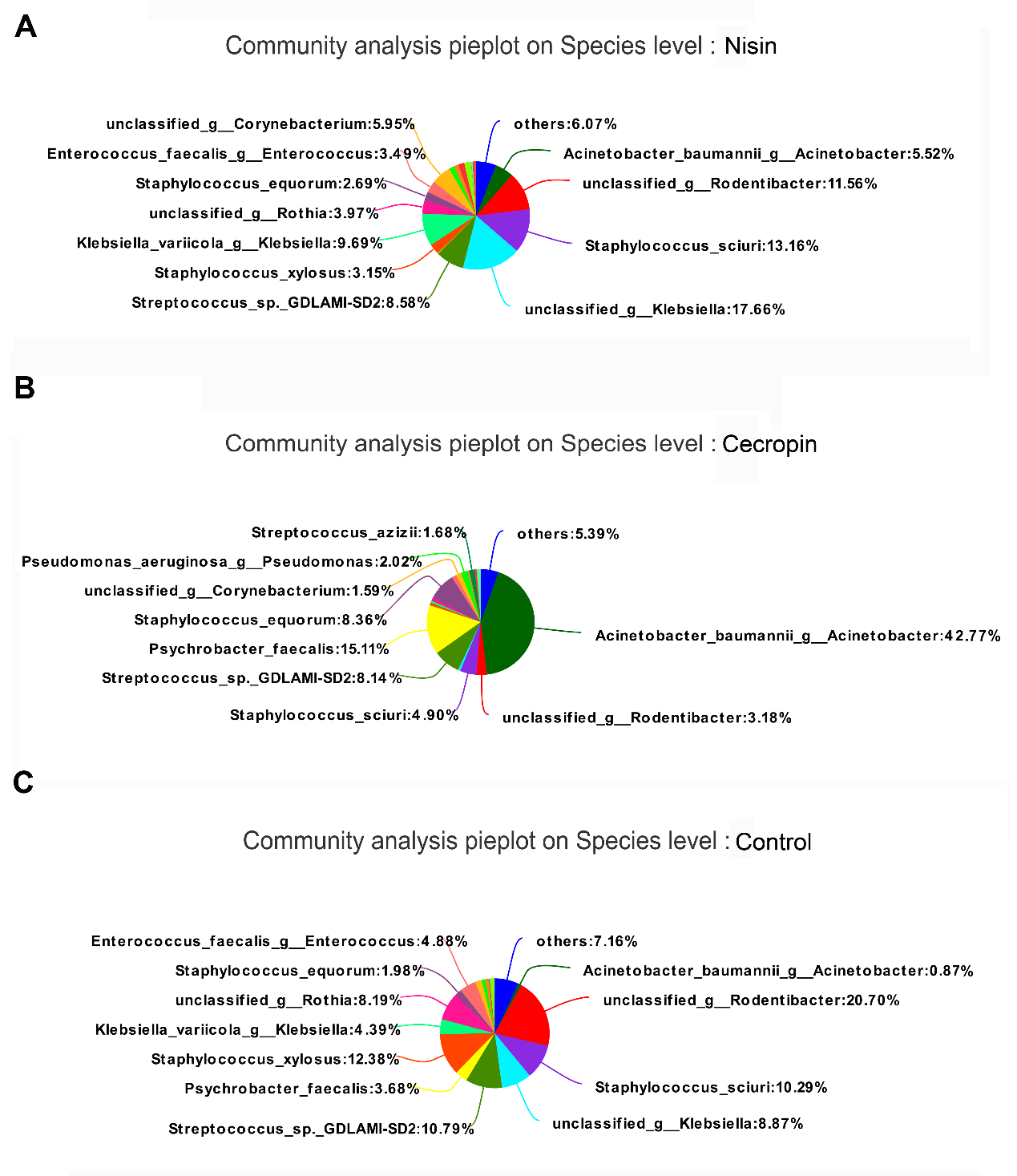


**Figure S1. Distribution of bacterial species among the three groups.** The pie diagrams show the bacterial composition of the nisin group (A), the cecropin group (B), and the control group (C). The relative abundances of the bacterial species in each group are shown. Others represent bacteria with less than 1% abundance at the species level.
